# Do feasibility studies contribute to, or avoid, waste in research?

**DOI:** 10.1371/journal.pone.0195951

**Published:** 2018-04-23

**Authors:** Ben Morgan, Jennie Hejdenberg, Saba Hinrichs-Krapels, David Armstrong

**Affiliations:** 1 National Institute for Health Research Central Commissioning Facility, Twickenham, United Kingdom; 2 Policy Institute, King’s College London, London, United Kingdom; 3 Department of Primary Care & Public Health Sciences, King’s College London, London, United Kingdom; University of Exeter, UNITED KINGDOM

## Abstract

In the context of avoiding research waste, the conduct of a feasibility study before a clinical trial should reduce the risk that further resources will be committed to a trial that is likely to ‘fail’. However, there is little evidence indicating whether feasibility studies add to or reduce waste in research. Feasibility studies funded by the National Institute for Health Research’s (NIHR) Research for Patient Benefit (RfPB) programme were examined to determine how many had published their findings, how many had applied for further funding for a full trial and the timeframe in which both of these occurred. A total of 120 feasibility studies which had closed by May 2016 were identified and each Principal Investigator (PI) was sent a questionnaire of which 89 responses were received and deemed suitable for analysis. Based on self reported answers from the PIs a total of 57 feasibility studies were judged as feasible, 20 were judged not feasible and for 12 it was judged as uncertain whether a full trial was feasible. The RfPB programme had spent approximately £19.5m on the 89 feasibility studies of which 16 further studies had been subsequently funded to a total of £16.8m. The 20 feasibility studies which were judged as not feasible potentially saved up to approximately £20m of further research funding which would likely to have not completed successfully. The average RfPB feasibility study took 31 months (range 18 to 48) to complete and cost £219,048 (range £72,031 to £326,830) and the average full trial funded from an RfPB feasibility study took 42 months (range 26 to 55) to complete and cost £1,163,996 (range £321,403 to £2,099,813). The average timeframe of feasibility study and full trial was 72 months (range 56 to 91), however in addition to this time an average of 10 months (range -7 to 29) was taken between the end of the feasibility study and the application for the full trial, and a further average of 18 months (range 13 to 28) between the application for the full trial and the start of the full trial. Approximately 58% of the 89 feasibility studies had published their findings with the majority of the remaining studies still planning to publish. Due to the long time frames involved a number of studies were still in the process of publishing the feasibility findings and/or applying for a full trial. Feasibility studies are potentially useful at avoiding waste and de-risking funding investments of more expensive full trials, however there is a clear time delay and therefore some potential waste in the existing research pathway.

## Introduction

Chalmers and Glasziou [1, 2] have pointed out the wastefulness of much research when relevant questions are not asked, the study design is inappropriate or results are either inaccurately reported or not made promptly available. These strictures have particular relevance for clinical trials where investment is often large and the clinical benefits more immediate than laboratory-based biomedical research. Indeed, one study of almost 8,000 cancer trials found that 20% failed to complete: a waste, not only in terms of funding, but also the time and commitment of the 48,000 patients involved [3]. Trials which ‘fail’ normally do so because they are unable to recruit the target number of patients. In recent years it has become increasingly common to conduct a preliminary ‘feasibility study’ to assess whether a full trial is likely to be successfully completed.

Feasibility studies ‘de-risk’ the funding of a full clinical trial. If the feasibility study shows that a full trial would be unlikely to meet the necessary patient recruitment/retention rates, intervention acceptability amongst other feasibility outcomes then the potential wasted cost of that trial is avoided. If, on the other hand, the feasibility study shows a full trial is likely to be successfully completed it reassures both funders and researchers that the cost and effort is likely to be worthwhile. Yet, while feasibility studies might avoid the wastefulness of funding trials that will not be successfully completed, they add costs, including the inevitable delay they introduce in completing any subsequent full trial. Do these costs of feasibility studies outweigh the subsequent waste they help avoid? The recent growth in number of feasibility studies has assumed the savings are worthwhile–but as yet without firm empirical evidence.

The National Institute for Health Research (NIHR) is a major funder of clinical trials in the UK (particularly through its Health Technology Assessment programme). Feasibility studies to see whether such trials are likely to be completed contribute to funding decisions; over the last decade the Research for Patient Benefit (RfPB) programme has funded over a hundred such studies.

Whether feasibility studies contribute to, or avoid, research waste was assessed in this study by investigating the outcomes of such studies funded by the RfPB programme.

## Methods

Feasibility studies that had completed by May 2016 were identified from the RfPB project database by searching for keywords, ‘feasibility’, ‘feasible’ and ‘pilot’. While there is some overlap between feasibility and pilot studies, recent guidance has proposed that pilot studies should be seen as a sub-set of feasibility studies [4]. Furthermore, a recent extension to the CONSORT statement to include pilot and feasibility studies will assist future reporting of such studies [5]. Nevertheless, for this study projects identified from the keyword search were scrutinised for indications that they were investigating whether a subsequent full trial would be feasible.

The principal investigator (PI) for each identified feasibility study was then sent a questionnaire asking them (a) to confirm their study was intended to assess the feasibility of a full trial, (b) whether the study had shown a full trial to be feasible, (c) whether they had applied for funding for such a trial and (d) whether they had published the results of their feasibility study. A reminder email was sent to PIs followed up by a telephone call for those who had not responded.

Responses were collated and examined in the context of the cost of the feasibility study findings for subsequent full trials, and in relation to the timeline from initial funding to (anticipated) trial completion.

## Results

**Fig 1:**
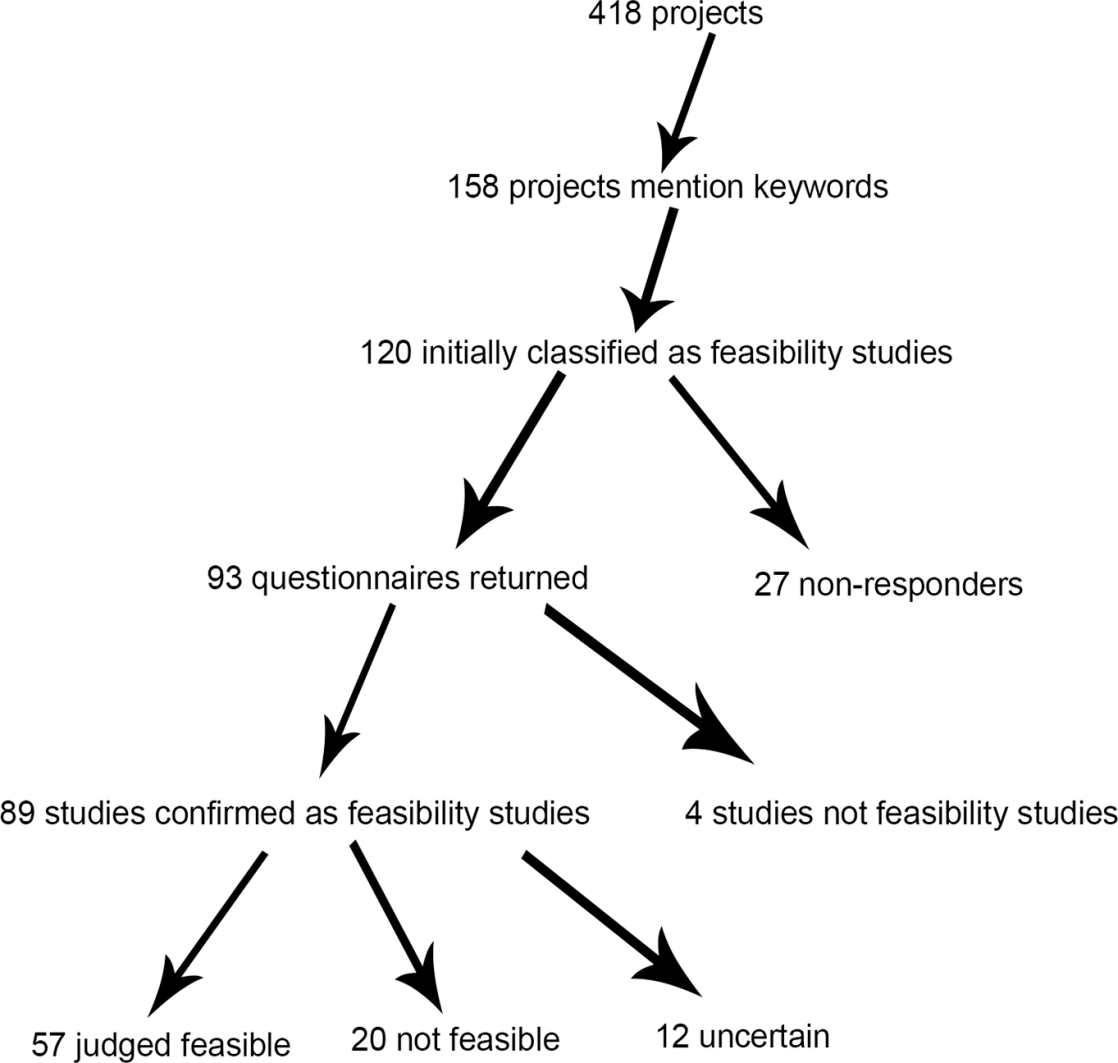
Number of projects studied.

Key word searches conducted on all 418 RfPB studies that had closed by May 2016 identified 158 unique studies. These studies were then reviewed (by BM and JH) and 120 were identified as being concerned with investigating feasibility parameters for a full trial. Questionnaires were sent to the principal investigators. Fig 1 shows the responses to the survey. Ninety-three responses were received; four reported not having conducted a feasibility study giving a valid response rate of 77% (89 responses suitable for analysis). Of those responding, 57 thought a full trial feasible, 20 that it was not and 12 were not sure.

The four responses stating that a feasibility study had not been conducted were due to one study being eventually conducted as a full trial, one study being a piece of developmental work prior to a feasibility study, and two studies where the PIs stated that their studies were not feasibility studies with a view to progress to a full trial.

A total of 27 of the PIs who were sent the questionnaire did not respond. Table 1 shows that the characteristics of the feasibility studies which responded were not significantly different from those which did not respond.

**Table 1:** Comparison between responders and non-responders.

	Responders (n = 93)	Non-responders (n = 27)
Feasibility study value (£)	218,291 (range: 72,031–326,830)	192,186 (range: 68,160–256,809)
Feasibility study duration (months)	31 (range: 18–50)	31 (range: 18–44)
Time since feasibility study ended (months)	31 (range: 2–81)	36 (range: 4–75)

An important source of research waste identified by Chalmers and Glasziou was the non- or delayed publication of results. Table 2 shows the proportion of feasibility studies in this sample that had published their results or were still intending to do so. Most respondents had published their results though about a third were still only intending to do so despite their study having completed on average over 14 months ago.

**Table 2:** Numbers of studies showing feasibility.

Is a full trial feasible?	Have already published the results (mean time since study end in months)	Still proposing to publish (mean time since study end in months)	Not proposing to publish	Non-response to this question
Judged feasible (n = 57)	39 (37)	13 (14)	1	4
Judged not feasible (n = 20)	10 (33)	7 (17)	1	2
Uncertain (n = 12)	3 (35)	8 (15)	0	1

The main purpose of a feasibility study is to ‘de-risk’ potential full trial funding. Ideally, therefore all studies in which a full trial was shown to be feasible should be on a trajectory to full trial funding. Table 3 shows that of the 57 studies where a full trial was judged feasible 15 had secured funding for further research (including one feasibility study leading to two full trials) including 12 full trials, two pilot studies and two pieces of further developmental research. A further 17 had tried to secure funding and been unsuccessful but were still trying while 17 reported that they had not yet applied but still intended to do so.

**Table 3:** Subsequent funding for feasibility studies.

Is a full trial feasible?	Full trial funded (mean/median time since study end in months)	Applied for full trial funding: unsuccessful but still trying (mean/median time since study end in months)	Applied for full trial funding: unsuccessful; now stopped (mean/median time since study end in months)	Not yet applied for funding but still intending (mean/median time since study end in months)	Not intending to apply for full trial funding (mean/median time since study end in months)	Non-response to this question
Judged feasible (n = 57)	15 (47/51)	17 (29/26)	4 (26/25)	17 (22/19)	4 (43/38)	0
Judged not feasible (n = 20)	0	0	0	6 (29/20)	14 (27/27)	0
Uncertain (n = 12)	0	1 (31)	1 (48)	6 (14/8)	3 (21/21)	1

Feasibility studies can avoid waste by preventing the funding of trials that would likely ‘fail’. The funding for the full trials obtained by the 12 successful feasibility study applicants was just over £1m per trial (mean £1,163,966, range £321,403 to £2,099,813) implying a rough ‘saving’ of £20-30m if all those which were either not feasible or uncertain if feasible (20 and 12 respectively) had applied directly for and been successful in getting full trial funding. The mean cost of the feasibility studies in this review was £219,048 (range £72,031 to £326,830).

**Fig 2:**
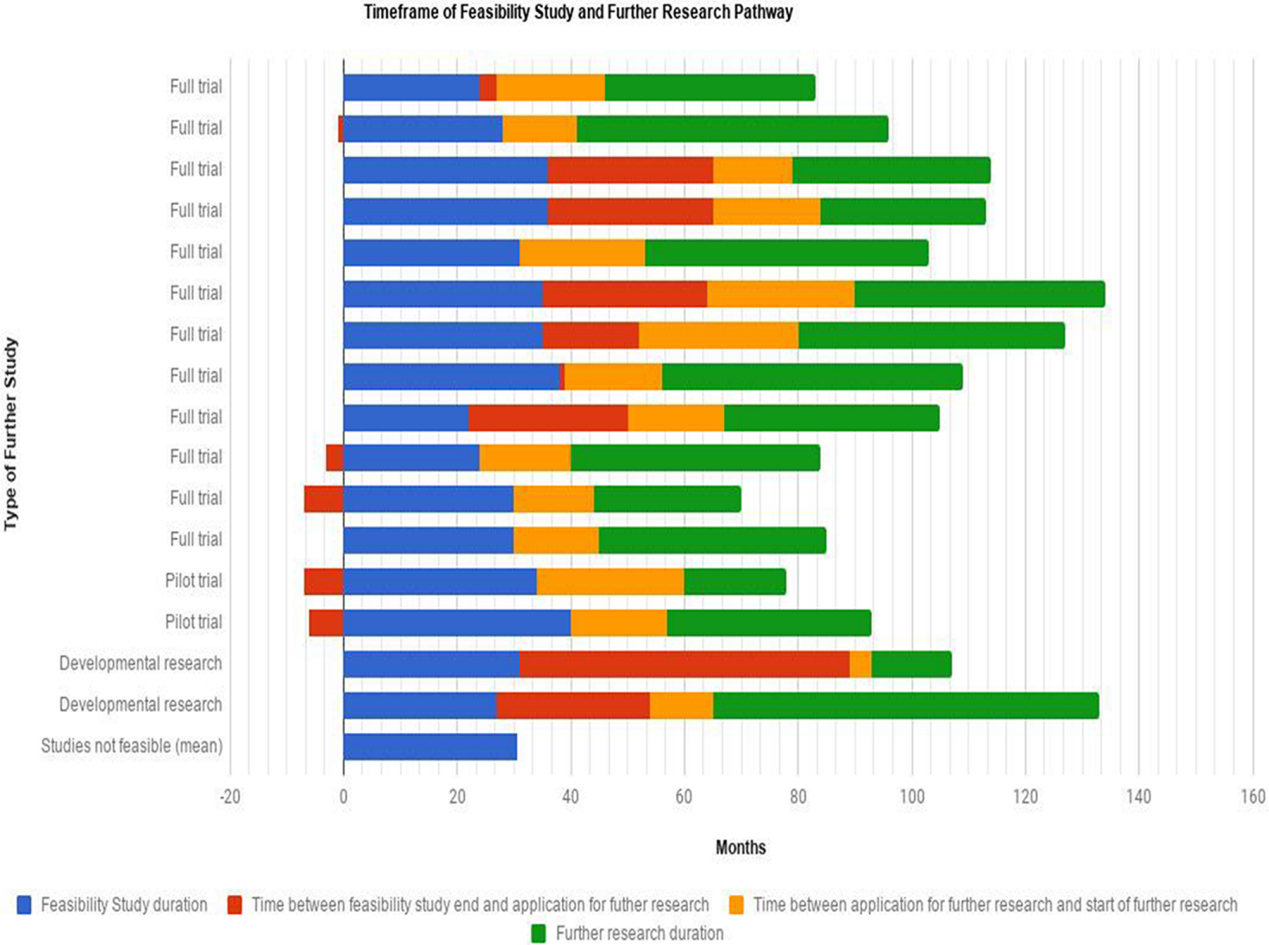
Duration of further research.

The timeline of the 15 feasibility studies that progressed along the pathway to full trials (though some of these involved further development work, including subsequent pilot trials) is shown in Fig 2 and Table 4. The feasibility studies lasted about three years but then many paused before proceeding for further funding. That application process and subsequent start delays added a further year or so to the timeline. The mean time trajectory was about eight years (assuming full trials complete to time) until final results that might benefit patients would be produced. The mean time between completing the feasibility study and applying for a full trial was 10 months (during which applicants might have had to wait for funding call dates) and, if successful, it took a further 18 months for the full trial to start.

**Table 4:** Mean of further research durations.

Type of further study	Feasibility Study duration (mean months)	Time between feasibility study end and application for further research (mean months)	Time between application for further research and start of further research (mean months)	Further research duration (mean months)
Full trial (n = 12)	31	10	18	41.5
Pilot trial (n = 2)	37	-6.5	21.5	27.5
Developmental research (n = 2)	29	42.5	7.5	37.5
Studies not feasible (n = 20)	31	n/a	n/a	n/a

Table 5 shows the funders which researchers applied to for the full trial. These sources are almost all separate programme funding streams within NIHR. It includes funders which either funded the full trial/further research (which may not necessarily have been the researchers first application) or the first application for a full trial which was unsuccessful. Out of a total of 38 researchers who had applied for further funding a total of 9 had submitted multiple applications to funders and a total of 19 researchers are still planning applications to funders.

**Table 5:** Applications for full trials.

Number of applications (number funded)	Funder
23 (7)	NIHR Health Technology Assessment (HTA)
5 (2)	NIHR Programme Grants for Applied Research (PGfAR)
4 (3)	NIHR Research for Patient Benefit (RfPB)
2 (1)	NIHR Efficacy and Mechanism Evaluation (EME)
2 (2)	NIHR Public Health Research (PHR)
2 (0)	Unknown
1 (1)	NIHR Health Services and Delivery (HS&DR)

## Discussion

About £19.5m was ‘invested’ in these 89 feasibility studies and they leveraged a further £16.8m with follow-on funding. Forty seven of the PIs said they were still intending to apply for the full trial and if such future applications were to be successful this latter figure would increase considerably. The 20 projects that reported negative and the 12 with ‘uncertain’ feasibility results represent savings by preventing ‘failed’ trials. With full trials costing about £1m each, this saving is considerable. However, whether all these clinical questions would have been judged as priorities [2]–especially three years after the initial study award–and therefore funded is difficult to judge.

The success rates in obtaining full trial funding for ‘positive’ feasibility studies, however, were not as high as might be expected for ‘de-risked’ trials. About half of positive feasibility studies had applied for funding but only half of these had been successful. According to the responses to our questionnaires, lack of success had not deterred many of these applicants and subsequent funding might yet be obtained. A further third had not applied but were still intending to. Even so, each of these feasibility studies that does not proceed to the next step represents some sort of wasted resource and opportunity.

Publication of the study findings should guide future research in the area and contribute value to the field. Yet publication figures are not encouraging with just over half having published so far. This publication rate is similar to that reported for full trials [6, 7, 8, 9, 10, 11] especially negative ones, though some funders report better results [12]. These figures might partly be explained by the difficulty in publishing the results of feasibility studies, however the appearance of more journals specialising in trial methods, such as *Pilot and feasibility studies*, may help this situation in future.

Finally, as Fig 2 shows, there are delays in generating conclusive results which may affect patients’ welfare. Feasibility studies added between 50–100% to the time of a full trial. Some of this delay was attributable to the research team taking time to apply. Almost half the feasibility studies that led to a full trial incurred delays of several months between the feasibility study finishing and the application for a full trial. This may depend on the nature of the uncertainty parameters the feasibility study was testing: some feasibility studies might have to wait until the very end of the study to submit an application while others can start preparing that application beforehand. The delays between a positive funding decision and a trial starting are likely to be influenced by regulatory hurdles and staff recruitment. It is not clear how far these can be further reduced.

When Chalmers and Glasziou identified waste in research they first described poor research questions and research design. Feasibility studies represent an attempt to get the question and design right prior to full funding so avoiding the trap of supporting projects that ask the wrong questions and/or not designed to be successfully completed. When feasibility studies demonstrate that a full trial is not feasible, they prevent waste. We have estimated that for an outlay of £19.5 million on feasibility studies in one programme this may have saved the wider NIHR at least £20 million. When a full trial is feasible, then the costs and benefits are more difficult to estimate, as the failure of many of the feasibility studies described here to proceed in a timely manner may represent another source of waste rather than waste avoidance. As Fig 2 shows, it takes about 5 years between applying for funding and getting the trial results (assuming extensions are not needed); a feasibility study adds another 3 years to this total. Feasibility studies might therefore save money but they do not save time. Yet, without prior feasibility studies research funding for some important clinical questions might be judged as too high risk, and therefore may not be funded at all. Feasibility studies can provide that reassurance and potentially avoid subsequent waste but at a cost in terms of the time it takes to get an answer to the question.

## Supporting information

S1 Fig Feasibility study questionnaire. (PDF)
