# Supplementary material for: Do feasibility studies contribute to, or avoid, waste in research?
Source: PLoS One. 2018 Apr 23;13(4):e0195951. doi: 10.1371/journal.pone.0195951 (PMC5912740; doi:10.1371/journal.pone.0195951)
Supplement: S1 Fig — (PDF) [file pone.0195951.s001.pdf]

Feasibility Study Survey

The Research for Patient Benefit (RfPB) programme is collecting data related to previously identified feasibility studies. The purpose of this review is to gain a greater understanding of the benefits of funding feasibility studies, and includes tracking the projects that included feasibility work. RfPB is interested in collecting information that will help the programme team better understand the trajectory that feasibility projects tend to take after the RfPB work is completed, particularly with regard to where researchers apply for further funding.

*Required

Project details

1. **Principal Investigator** *

2. **Have you undertaken an RfPB project that involved feasibility or pilot work that was intended to inform a full evaluation, if the study showed this to be feasible? Please refer to the NIHR definition of feasibility work for guidance when answering this question: http://www.nihr.ac.uk/CCF/RfPB/FAQs/Feasibility_and_pilot_studies.pdf** *

   Mark only one oval.

   - Yes *Skip to question 3.*
   - No *Skip to question 34.*

RfPB project that involved feasibility or pilot work was undertaken

3. **Please provide the Award Reference** *

RfPB project that involved feasibility or pilot work was undertaken

4. **Did the work undertaken within this award show that a full evaluation (e.g. a fully powered RCT) could be undertaken?**

   Mark only one oval.

   - Yes *Skip to question 5.*
   - No *Skip to question 16.*
   - Uncertain *Skip to question 5.*

RfPB work showed that a full evaluation would be feasible, or outcome was uncertain

5. **Has further funding been sought for a full evaluation based on the RfPB work?** *

   Mark only one oval.

   - Yes *Skip to question 6.*
   - No *Skip to question 12.*

Further funding has been applied for in order to undertake a full evaluation

6. **Please provide the date on which the application for further funding was submitted** *

7. **Was the application for further funding successful?** *
Mark only one oval.
- Yes *After the last question in this section, skip to question 10.*
- No *After the last question in this section, skip to question 25.*

8. **If yes, please provide the amount of funding awarded and duration**

9. **Please provide details of any applications for further funding, whether successful or otherwise. Please include funding organisation, date application was submitted, time and funding amount sought, reason for rejection if unsuccessful and Award Reference if successful.** *

Stop filling out this form.

Funding was obtained for full evaluation study based on the results of the RfPB feasibility work

10. **Has the full evaluation been completed?** *
Mark only one oval.
- Yes
- No

11. **If yes, please provide details of the outcome of this evaluation, and of any publications or outputs arising from the study.**

Skip to question 36.

Further funding has not been applied for in order to undertake a full evaluation, although results from RfPB study suggests a full evaluation would be feasible

12. **Please provide details of why further funding has not been sought, and whether or not there are any plans to apply for further funding in the future.** *

Skip to question 13.

Further funding has not been applied for in order to undertake a full evaluation, although results from RfPB study suggests a full evaluation would be feasible

13. **Have the results of the RfPB work been published?** *

Mark only one oval.

- Yes
- No

14. **If the work has been published, please provide details of the journal(s).**

15. **If the work has not yet been published, has it been submitted for publication? Please provide details of any journals that the work has been submitted to, including journals which have rejected the submission, and the reason for rejection.**

Skip to question 36.

RfPB work showed that a full evaluation would not be feasible

16. **Have the results of the RfPB work been published?** *
Mark only one oval.

- Yes
- No

17. **If the work has been published, please provide details of the journal(s).**

18. **If the work has not yet been published, has it been submitted for publication? Please provide details of any journals that the work has been submitted to, including journals which have rejected the submission, and the reason for rejection.**

19. **If the work has not been submitted for publication, please provide the rationale for this decision.**

20. **Has further funding been sought based on the RfPB work?** *
Mark only one oval.

- Yes *Skip to question 21.*
- No *Skip to question 22.*

Further funding has been sought based on the RfPB work

21. **Please provide details of any applications for further funding, whether successful or otherwise. Please include funding organisation, date application was submitted, time and funding amount sought, reason for rejection if unsuccessful and Award Reference if successful.** *

Skip to question 36.

Further funding has not been sought based on the RfPB work

22. **Are there any plans in place to seek further funding based on the work undertaken or the results obtained in the RfPB project?** *

Mark only one oval.

- Yes
- No

23. **If yes, please provide details of these plans, including which funder(s) will be approached, the likely value and duration of award sought, and the anticipated date that an application for funding will be made.**

24. **If no, please provide the rationale for not applying for any further funding based on the RfPB work.**

Skip to question 36.

Application for further funding based on the RfPB work has been unsuccessful

25. **Have the results of the RfPB work been published?** *

Mark only one oval.

- Yes
- No

26. **If the work has been published, please provide details of the journal(s).**

27. **If the work has not yet been published, has it been submitted for publication? Please provide details of any journals that the work has been submitted to, including journals which have rejected the submission, and the reason for rejection.**

28. **Are there any plans in place to seek further funding based on the work undertaken or the results obtained in the RfPB project?** *
Mark only one oval.

- Yes
- No

29. **If yes, please provide details of these plans, including which funder(s) will be approached, the likely value and duration of award sought, and the anticipated date that an application for funding will be made.**

30. **If no, please provide the rationale for not applying for any further funding based on the RfPB work.**

Skip to question 36.

RfPB work did not provide conclusive evidence to determine whether full evaluation would be feasible

31. **Have the results of the RfPB work been published?** *
Mark only one oval.

- Yes
- No

32. **If the work has been published, please provide details of the journal(s).**

33. **If the work has not yet been published, has it been submitted for publication? Please provide details of any journals that the work has been submitted to, including journals which have rejected the submission, and the reason for rejection.**

Skip to question 36.

Project did not involve feasibility work

Your project has been incorrectly identified as containing feasibility work intended to inform a larger evaluation. Apologies for any inconvenience this may have caused. Please provide the award details so that the records can be updated .

34. **Award Reference**

35. **Please let us know why you do not consider that the project constituted feasibility work, and provide any additional information that you**

Stop filling out this form.

Additional information

36. **Please provide any additional information regarding the outcomes of the RfPB work that may be useful, for example if it contributed to capacity building or fed into future work in a way that has not been captured within this survey.**

Powered by
Google Forms
